# PaleAle 6.0: Prediction of Protein Relative Solvent Accessibility by Leveraging Pre-Trained Language Models (PLMs)

**DOI:** 10.3390/biom15010049

**Published:** 2025-01-02

**Authors:** Wafa Alanazi, Di Meng, Gianluca Pollastri

**Affiliations:** 1 School of Computer Science, University College Dublin (UCD), D04 V1W8 Dublin, Ireland; di.meng@ucdconnect.ie (D.M.); gianluca.pollastri@ucd.ie (G.P.); 2 Department of Computer Science, College of Science, Northern Border University, Arar 73241, Saudi Arabia

**Keywords:** protein structure prediction, structural bioinformatics, bioinformatics, natural language processing, computational biology, deep learning

## Abstract

Predicting the relative solvent accessibility (RSA) of a protein is critical to understanding its 3D structure and biological function. RSA prediction, especially when homology transfer cannot provide information about a protein’s structure, is a significant step toward addressing the protein structure prediction challenge. Today, deep learning is arguably the most powerful method for predicting RSA and other structural features of proteins. In particular, recent breakthroughs in deep learning—driven by the integration of natural language processing (NLP) algorithms—have significantly advanced the field of protein research. Inspired by the remarkable success of NLP techniques, this study leverages pre-trained language models (PLMs) to enhance RSA prediction. We present a deep neural network architecture based on a combination of bidirectional recurrent neural networks and convolutional layers that can analyze long-range interactions within protein sequences and predict protein RSA using ESM-2 encoding. The final predictor, PaleAle 6.0, predicts RSA in real values as well as two-state (exposure threshold of 25%) and four-state (exposure thresholds of 4%, 25%, and 50%) discrete classifications. On the 2022 test set dataset, PaleAle 6.0 achieved over 82% accuracy for two-state RSA (RSA_2C) and 59.75% accuracy for four-state RSA (RSA_4C), with a Pearson correlation coefficient (PCC) of 77.88 for real-value RSA prediction. When evaluated on the more challenging 2024 test set, PaleAle 6.0 maintained a strong performance, achieving 79.74% accuracy in the two-state prediction and 55.30% accuracy in the four-state prediction, with a PCC of 73.08 for real-value predictions, outperforming all previously benchmarked predictors.

## 1. Introduction

The study of protein folding and function relies critically on the relative solvent accessibility (RSA) of amino acid residues, a property intrinsically linked to the spatial arrangement and packing of amino acids within the protein structure [1,2]. RSA, a one-dimensional (1D) property representing a protein’s relative exposure to a solvent, holds immense utility in predicting three-dimensional (3D) protein structures. Accurate RSA predictions contribute significantly to protein function analysis, hydration positioning, and overall structural insights [3]. While experimental methods for measuring RSA exist, they are resource-intensive and time-consuming, underscoring the necessity for efficient computational methods in the post-genomic era to keep pace with the rapidly growing collection of protein sequences [4].

Despite continuous updates from the Protein Data Bank (PDB) [5] through techniques like X-ray crystallography, the gap between known and unknown protein structures remains significant due to the challenges and expenses of experimental structure resolution [3]. This demand has spurred the development of various computational RSA predictors, many of which leverage machine learning to utilize protein sequence data effectively.

The prediction of RSA has evolved significantly, transitioning from traditional machine learning approaches to state-of-the-art deep learning models. Early methods relied on multi-class or binary classification frameworks using machine learning algorithms such as neural network-based regression, k-nearest neighbor [6], and support vector machines [7]. These methods achieved moderate accuracy, but their reliance on handcrafted features limited their scalability.

Existing RSA predictors are typically categorized as either discrete-valued or real-valued. Discrete predictors, such as RaptorX [8], ACCpro5 [9], SSpro/ACCpro 6 [10], PaleAle 5.0 [3], AcconPred [11], BMRSA [12], and IGPRED-MultiTask [13], segment RSA into categorized states (e.g., exposed, buried) using exposure thresholds (e.g., 25% and 50%). Despite their utility, these discrete methods lack the precision required for real-value RSA predictions, which limits their broader applicability.

Real-valued predictors, such as SPIDERS3 [14,15], SPIDER3-Single [16], NetSurfP-2.0 [17], NetSurfP-3.0 [18], DMVFL-RSA [4], SPOT-1D [19], and SPOT-1D-LM [20], have advanced RSA prediction by providing continuous RSA values per residue, thus addressing the limitations of discrete-valued approaches. Absolute solvent accessibility values from DSSP [14] are often normalized into RSA percentages, leveraging diverse calculation methods for different amino acids’ maximal exposure areas. RSA predictors further divide into template-based and ab initio models, depending on whether structural homology information is available. While template-based predictors often demonstrate a higher accuracy [21], they are constrained by the availability and appropriateness of the templates [22].

Recent advances in unsupervised deep learning methods, inspired by natural language processing [23], have provided innovative ways to extract information-rich features from protein sequences [20]. Models such as ProtTrans [24], trained on the UR50 dataset with transformer-based architectures like T5 and ESM-2 [25], trained on the UR90 dataset with 650 million parameters, have demonstrated substantial performance improvements.

In this study, we introduce PaleAle 6.0, a novel RSA predictor that builds upon the foundation established by PaleAle 5.0. While PaleAle 5.0 achieved success in discrete RSA classification, it lacked real-value RSA prediction capabilities, limiting its precision in applications requiring continuous exposure values. PaleAle 6.0 addresses this gap, providing flexible outputs in three formats: real values and two-state (2C) and four-state (4C) classifications.

In addition to supporting real-value predictions, PaleAle 6.0 introduces several advancements over PaleAle 5.0, including leveraging advanced embeddings from pre-trained language models (ESM-2) instead of evolutionary profiles and utilizing a convolutional bidirectional recurrent neural network (CBRNN) architecture for improved sequence representation. Furthermore, PaleAle 6.0 was trained on a larger, redundancy-reduced dataset of approximately 55 k proteins (up to 2022), enhancing its robustness and versatility. These updates resulted in approximately 3% improvement in performance compared to PaleAle 5.0 and established PaleAle 6.0 as a more comprehensive and accurate RSA predictor.

To develop this enhanced model, we conducted a comprehensive evaluation of deep learning architectures, including CNNs and RNNs, and their combination. Also, we assessed encoding methods (One-Hot, ProtTrans, and ESM-2) to optimize feature representation for protein sequences. Unlike other predictors which are limited to either continuous real-value or discrete classifications, PaleAle 6.0 multi-format predictions offer detailed RSA estimations alongside practical classifications for exposure thresholds (4%, 25%, and 50%). Additionally, by using a large, redundancy-reduced training dataset of approximately 500,000 proteins, with reductions of 80% and 30%, we optimized the model’s performance across benchmark datasets, advancing the robustness and versatility of RSA prediction. This marks an approximate 3% improvement in performance over its predecessor, PaleAle5.

## 2. Approach

This study aimed to develop an advanced approach for relative solvent accessibility (RSA) prediction, addressing key challenges in accurately predicting protein residue exposure. Our approach involves a thorough exploration and implementation of effective methods to create a specialized RSA predictor, with a focus on dataset generation, embedding techniques, model architecture, and training strategies.

Datasets and test sets were sourced from recent Protein Data Bank (PDB) entries. We evaluated several embedding techniques, including one-hot encoding and protein language model (PLM)-based embeddings, such as ESM-2 [18] and ProtTrans [17]. Simultaneously, we explored different neural network architectures to identify the optimal combination of network types and embedding methods that enhance RSA prediction accuracy.

A key component of this project was the deliberate exclusion of traditional multiple-sequence alignment (MSA) techniques [26], which are often time-consuming [1]. Instead, we leveraged PLMs, which generate dense, information-rich representations of protein sequences and serve as an efficient alternative to traditional evolutionary information for RSA prediction.

To capture both local and long-range dependencies in protein sequences, we employed advanced model architectures, including RNN, CNN, and CNN-BRNN combinations [2]. These architectures were selected based on their demonstrated effectiveness in related prediction tasks. Neural networks like RNN are particularly suitable for modelling complex sequence relationships, essential for RSA prediction, especially when trained on large datasets.

Our evaluation focused on three critical metrics for RSA prediction: Pearson correlation coefficient (PCC) for real-valued RSA predictions, accuracy (ACC), and F1-score for two- and four-state discrete RSA classes. PCC measured the precision of the continuous RSA values predicted for each residue, while ACC assessed the classification accuracy for residues labelled as exposed, intermediate, or buried at specified RSA thresholds. The F1-score, which combined precision and recall, provided a balanced measure of model performance, especially for imbalanced classes, offering deeper insights into the prediction quality for both two- and four-state RSA classifications. These metrics provided a comprehensive assessment of the model’s effectiveness in predicting solvent accessibility across exposure levels, offering deeper insights into protein structure and function.

This research aimed to create a reliable RSA predictor that tackles current challenges in data preparation, feature extraction, model design, and performance assessment. Utilizing cutting edge embedding methods and modern neural network frameworks, our objective was to develop an RSA predictor with high accuracy that effectively identified the intricate patterns associated with protein solvent accessibility.

## 3. Experiments

### 3.1. Datasets

The selection and preparation of datasets play a crucial role in machine learning tasks, particularly in protein prediction. For this study, we constructed datasets using the Protein Data Bank (PDB) [3], an accessible repository of protein structural data. Our initial dataset included 500,624 protein sequences from PDB entries released up until 16 November 2022, clustered using [27], at sequence identity thresholds of both 30% and 80%.

Regarding solvent accessibility calculations, solvent accessibility values were computed using the program in [28], based on experimentally resolved 3D structures in the PDB. For each amino acid *i,* the relative solvent accessibility (RSA) was calculated using the following formula:

(1) $$RSA_{i}=\frac{SA_{i}}{MAX_{i}}\times 100\%$$

where $SA_{i}$ is the solvent accessibility of residue *i* (in Å^2^) from DSSP, and $MAX_{i}$ is the maximum solvent accessibility for amino acid *i* (in Å^2^) type [29]. For the classification, amino acids were grouped into four RSA classes—[0–3%], [4–24%], [25–49%], and [50–∞%]—chosen to maintain balanced class distributions. For binary classification, the ranges [0–24%] and [25–∞%] were used.

#### 3.1.1. Training and Testing Set

We created training and test sets using clustering thresholds to increase sequence diversity and reduce redundancy:

- PDB 30%: Clustering at a 30% sequence identity yielded 25,600 sequences, with 4/5 reserved for training and 1/5 (5120 proteins) for the 2022 test set;
- PDB 80%: Clustering at an 80% identity produced 55,500 sequences, which were used for cross-validation training and to develop the final model.

An independent benchmark test set, the 2024 test set, was constructed from PDB entries released between 16 November 2022 and 20 July 2024, comprising 692 proteins clustered at a 30% sequence identity against the training set. Proteins with 10 or more undetermined amino acids were excluded to ensure data quality. Table 1 summarizes each dataset, including its source, purpose, and sequence count.

#### 3.1.2. Training and Evaluation Strategy

To ensure generalizability and robustness, we employed a five-fold cross-validation approach using the clustered datasets PDB 80% and PDB 30%. The PDB 80% dataset, consisting of 55,500 sequences clustered at an 80% identity threshold, was used as the primary source of training sequences for each fold. Meanwhile, the PDB 30% dataset, with 25,600 sequences clustered at a 30% identity threshold, was divided into five equal parts, each containing approximately 5120 sequences. In each fold of cross-validation, one part of the PDB 30% dataset served as the test set. To ensure that the training set contained no overlapping sequences with the test set, we used the MMseqs2 (13-45111+ds-2) Tool [30] to filter the PDB 80% dataset against the selected test set from PDB 30%, creating a unique training set for each fold. This approach allowed us to generate distinct training and test sets in each fold, enhancing the diversity and independence of the training data.

For final model benchmarking, we used the independent 2024 test set, which provided a comprehensive evaluation of our model’s performance on new, non-redundant protein sequences.

### 3.2. Sequence Embedding

To provide our RSA prediction model with meaningful sequence representations, we explored various embedding techniques, including one-hot encoding and protein language model (PLM)-based embeddings. These embeddings transform protein sequences into structured numerical inputs that can capture crucial information for accurate RSA prediction.

One-hot encoding is a foundational technique that represents each amino acid as a 21-element vector, where each position in the vector indicates a unique amino acid identity with a binary marker. This encoding preserves the distinct identity of each residue within the sequence, providing a straightforward yet effective representation for machine learning models.

PLM-based embeddings, such as those generated by ProtTrans and ESM-2, offer richer, more complex representations of protein sequences. ProtTrans, trained on the UniRef50 dataset, produces 1024-dimensional embeddings, encapsulating inter-residue relationships and capturing nuanced sequence features. ESM-2, trained on a large dataset, generates even higher-dimensional embeddings (1280 dimensions per residue), capturing deep structural and evolutionary information. Notably, ESM-2 supports sequences up to 1022 residues, which is generally suitable for most protein prediction tasks.

These PLM-based embeddings allow our RSA model to leverage advanced sequence features without relying on multiple-sequence alignment (MSA)-based evolutionary information, which is computationally intensive. By incorporating these dense, information-rich embeddings, our model is better positioned to recognize patterns linked to RSA, enhancing prediction accuracy.

### 3.3. Model Structure

With these embedding methods in place, we explored various model architectures optimized for RSA prediction, including recurrent neural networks (RNNs), convolutional neural networks (CNNs), and convolutional bidirectional recurrent neural networks (CBRNNs). Each architecture offers distinct advantages in processing protein sequences, allowing the model to capture both local and sequence-wide features that are important for RSA prediction.

Our primary model configuration is a convolutional bidirectional recurrent neural network (CBRNN), which combines the strengths of CNNs and bidirectional RNNs (BRNNs) to process both global dependencies and local patterns in protein sequences. The CBRNN model is particularly suited for RSA prediction, as it captures both context-dependent interactions and localized motifs associated with solvent exposure.

(1) Bidirectional Recurrent Neural Network (BRNN): The model begins with BRNN layers that process the protein sequence in both forward and backward directions. This bidirectional approach is essential for understanding interactions between amino acids that may be far apart in the sequence but are close in 3D space. By capturing these long-range dependencies, the BRNN provides the model with a comprehensive view of the sequence context, enhancing its ability to infer the RSA accurately.
(2) Convolutional Layers: Following the BRNN layers, convolutional layers are applied to further refine the sequence representation. CNNs are adept at recognizing local patterns, which is particularly valuable for RSA prediction, as residue accessibility often depends on neighboring amino acid configurations.
(3) These layers use a series of filters to extract high-level features, helping the model learn motifs that are associated with solvent exposure and structural properties. By combining BRNN and CNN layers, the CBRNN model effectively integrates both the global sequence context and localized features. This hybrid architecture allows PaleAle 6.0 to deliver enhanced RSA predictions by accurately modelling both the sequence-wide dependencies and structural motifs within protein sequences. The CBRNN’s unique ability to capture complex patterns makes it a powerful tool for RSA prediction, improving upon traditional methods by leveraging both spatial and sequential data within protein structures.

## 4. Training Process and Results

The training process for PaleAle 6.0 was conducted in two main phases, each focused on optimizing embedding methods, model architectures, and dataset configurations to maximize the RSA prediction accuracy.

### 4.1. Phase 1: Embedding and Architecture

In Phase 1, we assessed various embedding techniques (one-hot, ProtTrans, and ESM-2) and model architectures, exploring different combinations to identify the optimal configuration for RSA prediction. Using the PDB 30% dataset, we evaluated each setup across three RSA prediction tasks: two-class (RSA_2C), four-class (RSA_4C), and real-valued RSA. The results, summarized in Table 2, indicate that ESM embeddings consistently outperformed one-hot and ProtTrans embeddings across all tasks, achieving an accuracy (ACC) of 82.32% for RSA_2C, 59.51% ACC for RSA_4C, and a Pearson correlation coefficient (PCC) of 77.98 for real-valued RSA.

#### Model Architectures

The selected model structure, convolutional bidirectional recurrent neural network (CBRNN), was chosen for its ability to capture both sequence-wide dependencies and local patterns, which are essential for accurate RSA prediction. The model starts with bidirectional RNN layers, each with 40 hidden units per direction, allowing it to capture contextual dependencies across residues in both forward and backward directions. This is followed by a series of convolutional layers with Tanh activations, which extract localized sequence features crucial for RSA classification. The final output layers vary depending on the RSA format: sigmoid for two-class (binary), SoftMax for four-class (multi-class), and sigmoid for continuous real-valued RSA prediction. This hybrid structure enables PaleAle 6.0 to deliver robust predictions across binary, multi-class, and continuous RSA formats (Table S2).

**Figure 1:**
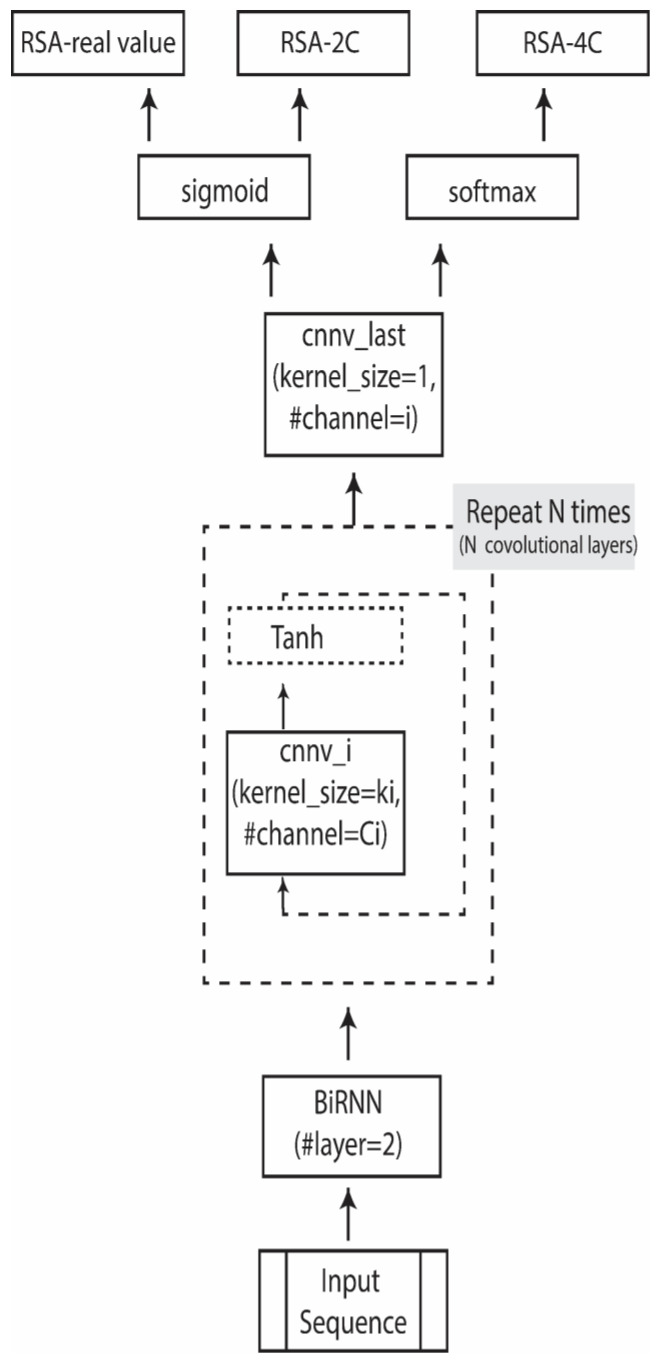
CBRNN structure for RSA prediction where N is the total number of convolutional layers, and *i* is the *i*th convolutional layer.

Among the tested architectures, the CBRNN model demonstrated superior performance compared to standalone RNNs and CNNs. Using ESM-2 embeddings, CBRNN achieved the highest performance with an accuracy of 82.32% for RSA_2C. Given its optimal performance, CBRNN was selected as the preferred architecture for further development in subsequent phases. We designed a consistent network structure for all three scenarios (RSA 2C, RSA-4C, and RSA-real), modifying only the activation function in the final layer to suit each specific case (Figure 1).

### 4.2. Phase 2: Final Training with Optimal Configuration

Phase 2 involved implementing the optimal configuration identified in Phase 1—ESM embeddings with the CBRNN architecture—on the larger PDB:80% dataset to develop the final RSA predictors. Training on this extended dataset led to further performance improvements, achieving an accuracy (ACC) of 82.48% for RSA_2C, 59.60% ACC for RSA_4C, and a PCC of 77.88 for real-valued RSA (Table 3).

To ensure robustness, we conducted five-fold cross-validation on the PDB:80% dataset. The results demonstrated consistent performance across folds, with an average accuracy of 82.56% for RSA_2C, 59.75% for RSA_4C, and an average PCC of 78.09 for real-valued RSA (Supplementary Table S1). These validation strategies align with the principles outlined by Greener et al., which emphasize the importance of unbiased evaluation and proper dataset partitioning to ensure reliability and reproducibility in supervised machine learning models [31].

The two-phase training process highlights the effectiveness of ESM embeddings and the CBRNN architecture for RSA prediction. The Phase 1 results indicated that the ESM-2 embeddings outperformed other methods across all RSA prediction formats, while Phase 2 confirmed that the model generalized well on a larger dataset. Five-fold cross-validation further validated the model’s stability and reliability, with consistent performance across folds, establishing PaleAle 6.0 as a robust tool for RSA prediction across binary, multi-class, and continuous formats.

#### 4.2.1. Ensemble Predictors in PaleAle 6.0

To enhance the accuracy and robustness of relative solvent accessibility (RSA) predictions, PaleAle 6.0 employs an ensemble modelling approach, integrating bidirectional recurrent neural networks (BRNNs) with convolutional neural networks (CNNs). This ensemble consists of fifteen models derived from a five-fold cross-validation process across the three RSA prediction tasks: binary classification (RSA_2C), four-class classification (RSA_4C), and real-valued RSA prediction. Each fold generates three models—one for each prediction type—resulting in a total of fifteen models. These models are trained on protein datasets with embeddings generated through ESM-2, capturing both sequence-wide dependencies and local structural patterns. By combining the strengths of BRNNs and CNNs, this ensemble approach not only improves generalization and prediction accuracy but also enhances model reliability, making PaleAle 6.0 a robust and versatile tool for RSA prediction across binary, multi-class, and continuous formats. This comprehensive approach positions PaleAle 6.0 as an advanced solution for practical applications in structural biology and protein modelling.

#### 4.2.2. Performance Comparison

We evaluated PaleAle 6.0 against several prominent RSA prediction models—NetSurfP-2.0, NetSurfP-3.0, SPOT-1D-LM, and the previous version of PaleAle (5.0)—using a 2024 benchmark test set of 692 proteins. The performance metrics included accuracy (ACC) and the F1 score for two- and four-class RSA predictions and the Pearson correlation coefficient (PCC) for the real-valued RSA. Results from the 2024 test set demonstrated PaleAle 6.0’s competitive performance, particularly for two- and four-class predictions, with an RSA_2C accuracy of 79.74% and an RSA_4C accuracy of 55.30%. These results significantly surpass those of PaleAle 5.0, which achieved 77.00% for RSA_2C and 51.54% for RSA_4C. The results are detailed in Table 4.

## 5. Discussion

The development and evaluation of PaleAle 6.0 underscore its effectiveness as a tool for predicting relative solvent accessibility (RSA) in proteins. By leveraging ensemble modelling and protein language model (PLM)-based embeddings from ESM-2, PaleAle 6.0 achieves strong performance across binary, multi-class, and continuous RSA prediction tasks. In this section, we discuss the model’s performance insights, key strengths, and implications and provide detailed comparisons with previous predictors, including results from both the 2022 and 2024 test sets.

The ensemble-based approach in PaleAle 6.0, which leverages PLMs like ESM-2 rather than traditional sequence alignments, demonstrates high accuracy while bypassing dependency on evolutionary information. The model’s performance across continuous, two-class, and four-class RSA predictions reveals distinct strengths in each predictive format.

Regarding real-valued RSA, the model achieves robust correlations with true RSA values, with Pearson correlation coefficients (PCCs) of 77.88 on the 2022 test set and 73.08 on the 2024 test set, outperforming NetSurfP-2.0 and NetSurfP-3.0, which obtained 68.60 and 69.22 PCC, respectively. This consistency in high PCC values highlights PaleAle 6.0’s strong performance in real-valued RSA predictions, making it particularly well-suited for applications requiring precise, continuous RSA distributions.

Regarding two-class RSA classification, PaleAle 6.0 achieves impressive accuracy in binary RSA classification, with accuracy (ACC) scores of 82.56% on the 2022 test set and 79.74% on the 2024 test set. This surpasses SPOT-1D-LM’s performance (78.34%), as well as NetSurfP-3 (77.35%) and NetSurfP-2 (77.17%). The high accuracy in two-class RSA classification underscores PaleAle 6.0’s capability to reliably distinguish between exposed and buried residues.

Regarding four-class RSA classification and prediction task, PaleAle 6.0 maintains a competitive accuracy of 59.75% on the 2022 test set and 55.30% on the 2024 test set, showing resilience despite the inherent difficulty of multi-class RSA predictions. This performance highlights PaleAle 6.0’s robustness in capturing nuances across multiple exposure states, which can be particularly useful for detailed structural applications.

In summary, the ensemble-based model in PaleAle 6.0, with its use of PLM-derived embeddings and targeted training across multiple RSA formats, delivers high accuracy and consistency across diverse RSA prediction tasks. This establishes PaleAle 6.0 as a flexible and reliable predictor, suitable for both research and practical applications in structural biology and protein modelling.

## 6. Conclusions

PaleAle 6.0 represents a significant advancement in predicting relative solvent accessibility (RSA) across binary, multi-class, and continuous formats. By integrating an ensemble of convolutional bidirectional recurrent neural networks (CBRNNs) with protein language model (PLM)-based embeddings, PaleAle 6.0 achieves high accuracy and robustness, outperforming existing models such as NetSurfP-2.0, NetSurfP-3.0, and the previous version, PaleAle 5.0. The use of ESM-2 embeddings allows PaleAle 6.0 to capture complex sequence dependencies, providing an alignment-free alternative to traditional evolutionary information-based approaches. This advancement highlights PaleAle 6.0’s capability for reliable and efficient RSA predictions that are well-suited for large-scale applications in structural biology.

Our findings demonstrate PaleAle 6.0’s strong performance across all RSA prediction formats, with notable accuracy in real-valued RSA predictions and multi-class classification tasks. The ensemble approach further enhances stability and accuracy, reducing variance across predictions and establishing PaleAle 6.0 as a robust tool for research and applied fields requiring precise RSA predictions. By training specifically for four-class RSA and leveraging diverse embeddings, PaleAle 6.0 captures nuanced distinctions across exposure states, making it a versatile predictor in the study of protein structure and function. Beyond accuracy, PaleAle 6.0 offers key advantages over direct structure prediction methods. RSA predictions are computationally less intensive and provide residue-level insights into solvent exposure, which are critical for understanding protein folding, stability, and function. PaleAle 6.0 is publicly available on GitHub https://github.com/WafaAlanazi/PaleAle6.git (accessed on 1 November 2024).

## Figures and Tables

**Table 1:** RSA dataset information.

Dataset	Seq Num	Strategy
PDB 30% (Train)	20,535	Blast 30% identity clustering
PDB 80% (Train)	55,500	Blast 80% identity clustering
2022 Test Set	5130	Blast 30% identity clustering
2024 Test Set	692	Blast 30% identity clustering

**Table 2:** Results of Phase 1 using the PDB 30% dataset, detailing the RSA_2C, RSA_4C, and real-valued RSA performance for each embedding method.

Predictors	One Hot	ProtTrans	ESM-2
RSA_2C	72.42% ACC	82.28% ACC	82.32% ACC
RSA_4C	44.93% ACC	59.29% ACC	59.51% ACC
RSA_ real value	57.50 PCC	77.63 PCC	77.98 PCC

**Table 3:** Results of Phase 2 on the PDB:80% dataset, with the final RSA_2C, RSA_4C, and real-valued RSA performance for ESM-2 embeddings.

Predictors	ESM
RSA_2C	82.48% ACC
RSA_4C	59.60% ACC
RSA_ real value	77.88% PCC

**Table 4:** Performance comparison on the 2024 test set.

Predictors	RSA_2C (ACC)	F1	RSA_4C (ACC)	F1	RSA_Real Value (PCC)
NetSurfP-2	77.17%	0.78			68.60%
NetSurfP-3	77.35%	0.78			69.22%
SPOT-1D-LM	78.34%	0.79			70.50%
PaleAle 5.0	77.01%	0.76	51.54%	0.51	-
PaleAle 6.0 (PDB 30%)	79.54%	0.79	55.04%	0.53	72.83%
PaleAle 6.0 (PDB 80%)	79.74%	0.79	55.30%	0.54	73.08%

## Data Availability

PaleAle 6.0 is publicly available on GitHub (https://github.com/WafaAlanazi/PaleAle6.git (accessed on 1 November 2024)).

## Supplementary Materials

The following supporting information can be downloaded at: https://www.mdpi.com/article/10.3390/biom15010049/s1.
