# Supplementary material for: PaleAle 6.0: Prediction of Protein Relative Solvent Accessibility by Leveraging Pre-Trained Language Models (PLMs)
Source: Biomolecules. 2025 Jan 2;15(1):49. doi: 10.3390/biom15010049 (PMC11764203; doi:10.3390/biom15010049)
Supplement: Supplementary file 1 [file biomolecules-15-00049-s001.zip › biomolecules-3370323-Supplementary.pdf]

Tabel S1, Detailed results of five-fold cross-validation in Phase 2 using PDB:80%, showing performance consistency.

Folds	RSA_2C(ACC)	RSA_4C(ACC)	RSA_ real Value(PCC)
Fold1	82.48%	59.60%	77.88 %
Fold2	82.52%	59.75%	78.09%
Fold3	82.52%	59.78%	78.06%
Fold4	82.70%	59.96%	78.28 %
Fold5	82.57%	59.68%	78.20%
**Average**	**82.56%**	**59.75%**	**78.10 %**

Tabel S2, Model structure of CBRNN; "i" in "Activation Function" refers to Sigmoid for 389 RSA-2C/realValues and Softmax for RSA-4C, the output channels in the last layer are written as (1-4), where 1 corresponds to RSA-2C/realValues and 4 corresponds to RSA-4C.

BRNN Layer

#layer	Layer Type	Input Size	Hidden Size	Output Size	Bidirectional	Number Layers
1	RNN	#features	40	80(40*2)	YES	2

CNN Layer

#layer	#in channel	#out_channel	Kernel size	stride	padding	Activation function
1	80	10	7	1	3	Tanh
2	10	8	3	1	1	Tanh
Last	8	(1-4)	1	1	0	*i*
